# Resistance to HSP90 inhibition involving loss of MCL1 addiction

**DOI:** 10.1038/onc.2015.213

**Published:** 2015-06-22

**Authors:** S Busacca, E W P Law, I R Powley, D A Proia, M Sequeira, J Le Quesne, A Klabatsa, J M Edwards, K B Matchett, J L Luo, J H Pringle, M El-Tanani, M MacFarlane, D A Fennell

**Affiliations:** 1 Department of Cancer Studies, Cancer Research UK Leicester Centre, University of Leicester, Leicester, UK; 2 MRC Toxicology Unit, Leicester, UK; 3 Synta Pharmaceuticals Corp., Lexington, MA, USA; 4 Division of Cancer Studies, King's College London, London, UK; 5 Centre for Cancer Research and Cell Biology, Queen's University Belfast, Belfast, UK; 6 Institute of Cancer Therapeutics, School of Life Sciences, University of Bradford, Bradford, UK

## Abstract

Inhibition of the chaperone heat-shock protein 90 (HSP90) induces apoptosis, and it is a promising anti-cancer strategy. The mechanisms underpinning apoptosis activation following HSP90 inhibition and how they are modified during acquired drug resistance are unknown. We show for the first time that, to induce apoptosis, HSP90 inhibition requires the cooperation of multi BH3-only proteins (BID, BIK, PUMA) and the reciprocal suppression of the pro-survival BCL-2 family member MCL1, which occurs via inhibition of STAT5A. A subset of tumour cell lines exhibit dependence on MCL1 expression for survival and this dependence is also associated with tumour response to HSP90 inhibition. In the acquired resistance setting, MCL1 suppression in response to HSP90 inhibitors is maintained; however, a switch in MCL1 dependence occurs. This can be exploited by the BH3 peptidomimetic ABT737, through non-BCL-2-dependent synthetic lethality.

## Introduction

Targeting the molecular chaperone heat-shock protein 90 (HSP90) is an attractive therapeutic strategy for treating cancer. HSP90 is essential for the maturation of client proteins, and its inhibition leads to client misfolding, ubiquitination and proteasomal degradation.^1^ Consequently, HSP90 inhibition is pleiotropic in its targeting, effectively inhibiting cancer networks.^2, 3, 4, 5^ The mechanisms underpinning resistance are poorly understood. HSP90 inhibition efficiently induces cancer cell apoptosis and may be selective to chaperone-dependent oncogenic drivers such as EML4-ALK.^6^ Different variants of the EML4-ALK fusion protein exhibit different stability and sensitivity to HSP90 inhibition^7^ and our recent data suggest that specific EML4-ALK variants exhibit differential sensitivity to HSP90 inhibition-mediated ubiquitination and degradation, owing to their TAPE domain structure.^8^ Cullin-RING E3 ubiquitin ligase Cullin-5 has an important role in mediating the HSP90 inhibitor 17-AAG-induced degradation of driver oncogenes that are HSP90 clients. Suppression of Cullin-5 has been proposed as a mechanism of acquired resistance in epidermal growth factor receptor-mutant tumours.^9^ The alteration of the expression of other heat-shock proteins, such as HSP70 and HSP27, is an intrinsic mechanism of resistance that can occur as a result of a compensatory response to protect cancer cells from stress insults.^10, 11^ Rapid drug metabolism has also been correlated to a reduction of the response to HSP90 inhibitors. UGT1A (UDP glucuronosyltransferase 1 family, polypeptide A complex locus) levels have been proposed as a predictive biomarker for response to resorcinolic HSP90 inhibitors,^12, 13^ whereas a reduced expression of NQO1 (NAD(P)H dehydrogenase quinone 1) has been shown to mediate resistance to 17-AAG and other geldanamycin analogues.^14^ Resistance to HSP90 inhibition has been associated with point mutations in the N-domain of *Humicola fuscoatra* and *S. hygroscopicus*. However, in eukaryotic cells a mutation in the ATP-binding site of HSP90 would be incompatible with the survival of the cell.^15^ The hyper activation of survival pathways and alteration of the apoptotic response^16^ have been shown to have an important role in drug resistance.

The mode of activation of apoptosis following HSP90 inhibition is poorly defined. The canonical mitochondrial apoptosis pathway is induced by the multidomain proapoptotic BCL-2 family members BAX and BAK,^17^ which control mitochondrial outer membrane permeabilization (MOMP).^18, 19^ MOMP is governed by the interplay of the anti-apoptotic Bcl-2 family proteins, which interact with BAX and BAK to prevent oligomerization and MOMP, and the pro-apoptotic BH3-only proteins, which can trigger apoptosis by direct or indirect interaction with BAX and BAK.^20, 21^ These sentinels also couple the mitochondrial apoptosis pathway to damage in distinct cellular compartments.^22, 23^

BAX has been shown to be required for induction of cell death by 17-AAG in human colon carcinoma cells *in vitro* and *in vivo*;^24^ however, the complement of essential BH3-only proteins is poorly characterized.

Using functional genetics, we have dissected the BCL-2 family members required for activation of mitochondrial apoptosis. MCL1 downregulation persists in the acquired resistance setting, albeit in the context of a switch in MCL1 dependence. This allows pharmacological exploitation to revert the HSP90 inhibitor-resistant phenotype via combination with a BH3-peptidomimetic.

## Results

### Apoptosis induced by inhibition of HSP90 requires the mitochondrial pathway

**Figure 1:**
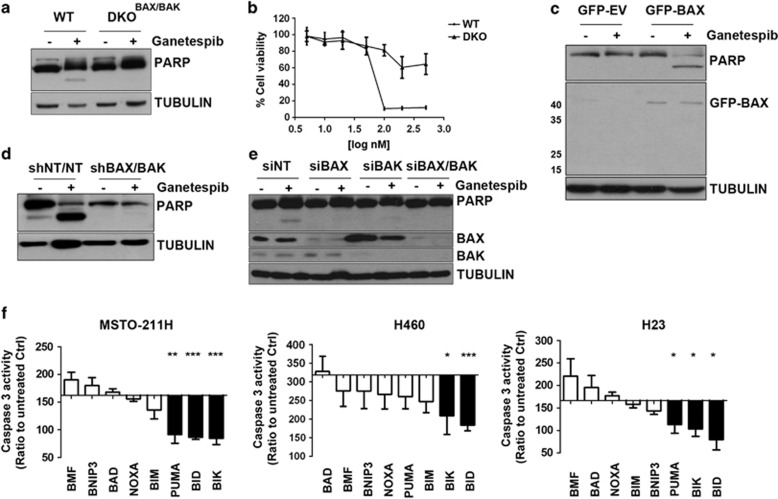
Requirement for functional mitochondria for ganetespib-induced apoptosis. (**a**) WT and DKO^BAX/BAK^ cells were treated with ganetespib 200 nm for 48 h. PARP cleavage was measured by western blot. (**b**) Viability was assessed by MTT assay after 72 h. WT: IC_50_=67.77 nm; DKO^BAX/BAK^ IC_50_>500 nm. (**c**) BAX was transiently overexpressed in DKO^BAX/BAK^ cells and 24 h post-transfection cells were treated with ganetespib 200 nm for a further 48 h. BAX expression and PARP cleavage were analysed by western blot. (**d**) MSTO-211H cells were transfected with siNT, siBAX, siBAK and the combination of siBAX and siBAK. Twenty-four hours following transfection, cells were treated with ganetespib for a further 48 h. (**e**) H460 stably expressing shRNA targeting BAX and BAX was treated with ganetespib 200 nm for 48 h and PARP cleavage analysed. (**f**) MSTO-211H, H460 and H23 have been transfected with siRNAs targeting BH3-only protein. Twenty-four hours after transfection cells have been treated with ganetespib 200 nm for further 48 h and Caspase 3 activity measured. Data were normalized to siNT control (MSTO-211H: siBID ***P=0.0001 siBIK ***P=0.0007; siPUMA **P=0.0023; H460: siBID ***P=0.0006 siBIK *P=0.0163; H23: siBID *P=0.0123 siBIK *P=0.0242; siPUMA *P=0.0481).

Resistance to HSP90 inhibition was observed in mouse embryonic fibroblasts harbouring homozygous deletion of BAX and BAK (DKO^BAX/BAK^), compared with wild-type (WT) cells (Figures 1a and b, Supplementary Figure S1A and B). Re-expression of exogenous BAX in DKO^BAX/BAK^ cells restored sensitivity (Figure 1c). Both stable^25^ and transient silencing of BAX and/or BAK rescued cells from HSP90 inhibition-induced apoptosis (Figures 1d and e, Supplementary Figure S1C and D), providing evidence for apoptosis induction via the intrinsic apoptotic pathway. Caspase 8 activation occurred in a BAX/BAK-dependent manner, further implicating an amplification loop via the mitochondria (Supplementary Figure S1E).

We utilized a focused siRNA panel to identify the complement of critical proapoptotic BH3-only proteins required for HSP90 inhibitor-induced apoptosis. Silencing of the activating BH3-only protein BID^26^ and pro-survival BCL-2 family inhibitor BIK^21^ significantly inhibited caspase 3 activation in all the cell lines analysed. PUMA induction was required for apoptosis in MSTO-211H and H23 cells only (Figure 1f, Supplementary Figure S2A–C). The activating BH3-only protein BIM,^21^ previously reported to be a critical mediator of HSP90 inhibition-induced apoptosis,^27^ was not found to be required (Supplementary Figure S2D).

The effect of silencing BID, BIK and PUMA on the response to ganetespib was confirmed with additional siRNA sequences (Supplementary Figure S2E).

### HSP90 inhibition transcriptionally suppresses MCL1 via STAT5A inhibition

**Figure 2:**
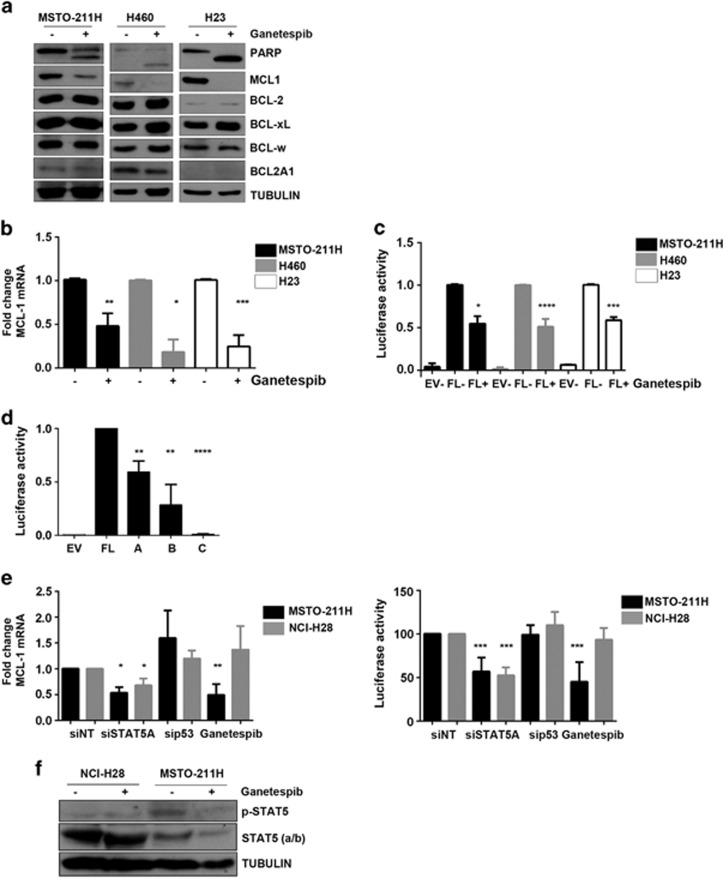
MCL1 is transcriptionally regulated by ganetespib. (**a**) MSTO-211H, H460 and H23 have been treated with ganetespib 200 nm for 48 h. PARP cleavage and the expression of the pro-survival BCL-2 family members have been assessed by western blot. (**b**) MCL1 mRNA expression was evaluated by qRT-PCR on RNA extracted from MSTO-211H, H460 and H23 cells treated for 48 h with ganetespib 200 nm. Data were normalized to untreated control (MSTO-211H: **P=0.0033; H460 *P= 0.0150; H23: ***P=0.0006). (**c**) The MCL1 promoter activity was measured by a luciferase reporter assay in MSTO-211H, H460 and H23 cells transfected with pGL2 basic (EV) or pGL2-MCL1 (FL) and then treated with ganetespib 200 nm for 24 h. Data were normalized to untreated full-length (FL) control (MSTO-211H: *P=0.0119; H460 ****P<0.0001; H23: ***P=0.0010). (**d**) MSTO-211H cells were transfected with three fragments of the promoter and treated with ganetespib 200 nm for 24 h. The MCL1 promoter activity was measured by a reporter assay. Data were normalized to untreated FL control (Fragment A: **P=0.0031; Fragment B **P=0.0077; Fragment C: ****P<0.0001). (**e**) The effect of siSTAT5A and sip53 on MCL1 mRNA expression and MCL1 promoter activity was measured by qRT-PCR on RNA extracted from MSTO-211H and NCI-H28 cells transfected for 24 h or luciferase reporter assay. Cells treated with ganetespib 200 nm were used as a positive control. Data were normalized to untreated control (qRT-PCR: MSTO-211H, siSTAT5A: *P=0.03977, sip53: n.s.; NCI-H28, siSTAT5A: *P=0.0439, sip53: n.s. Luciferase assay: MSTO-211H, siSTAT5A: ***P<0.0001, sip53: n.s.; NCI-H28, siSTAT5A: ***P=0.0003, sip53: n.s.). (**f**) The effect of ganetespib on STAT5 was measured by western blot.

To determine whether HSP90 inhibition modifies the expression of pro-survival BCL-2 family proteins to promote apoptosis, we examined the expression of BCL-2, BCL-xL, BCL-w, MCL1 and BCL2A1 pre- and post-HSP90 inhibition, and identified MCL1 downregulation as the sole modification across this pro-survival protein repertoire (Figure 2a, Supplementary Figure S3A).

Caspase-dependent cleavage of MCL1 did not account for its downregulation (Supplementary Figure S3B), and MCL1 mRNA levels were reduced following HSP90 inhibition as evidenced by qPCR and MCL1 promoter activity (Figures 2b and c). To identify possible MCL1-associated transcription factors, we generated three truncation mutants of the promoter region in the MCL1 luciferase reporter, called A, B and C (Supplementary Figure S3C). The shorter truncation mutants (A and B) were responsible for lower luciferase reporter activity, with the smallest fragment C having an activity similar to the empty vector (Figure 2d).

Using PROMO prediction software to identify putative transcription factor binding sites, a binding site for the HSP90 client STAT5A was predicted in a region present in fragments A and B but not C. A binding site for the HSP90 client p53 was also predicted but in multiple regions across the full-length promoter sequence.

We studied the effect of the silencing of both STAT5A (Supplementary Figure S3D) and p53 (Supplementary Figure S3E) in MSTO-211H and NCI-H28. RNAi silencing of STAT5A resulted in a significant reduction of MCL1 mRNA levels and MCL1 promoter activity in MSTO-211H cells as well as NCI-H28 cells, whereas p53 RNAi had no effect. (Figure 2e, Supplementary Figure S4A). The extent of the effect obtained with silencing of STAT5A was comparable to that achieved with ganetespib in MSTO-211H. In contrast, no effect on MCL1 expression or promoter activity was observed in NCI-H28 cells following treatment with ganetespib. We therefore hypothesized a lack of activity of the drug in inhibiting the interaction between HSP90 and STAT5, in NCI-H28 cells. Indeed, the treatment inhibited STAT5 in MSTO-211H, but not in NCI-H28 cells (Figure 2f).

### Sensitivity to HSP90 inhibition correlates with MCL1 dependence

**Figure 3:**
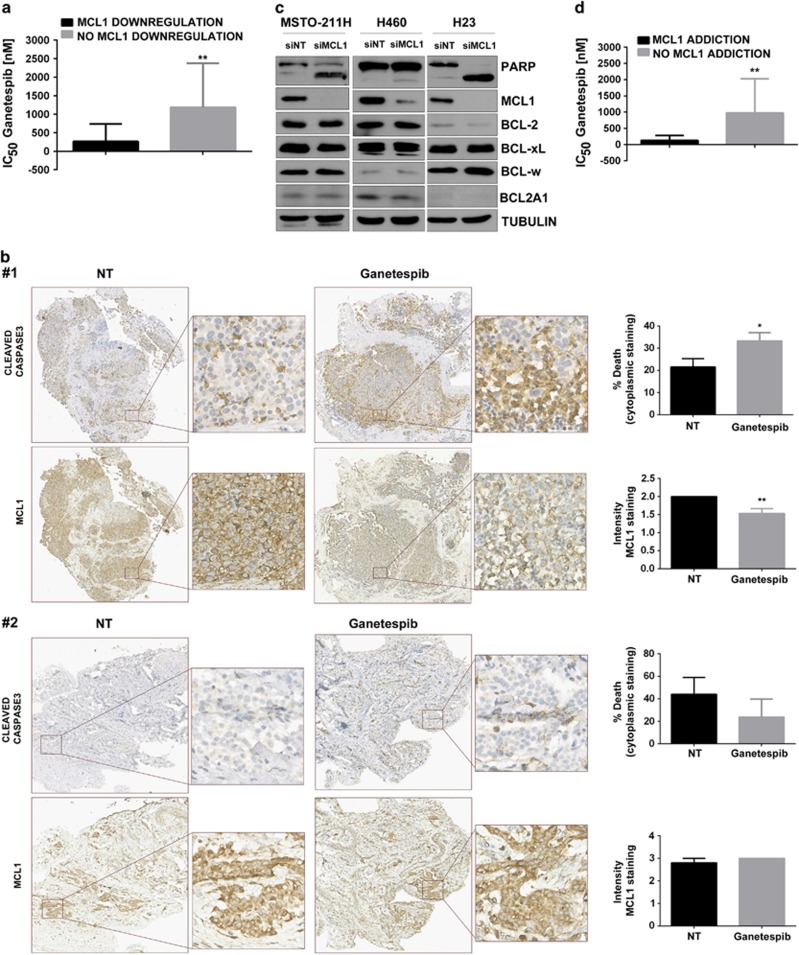
MCL1 suppression and MCL1 addiction correlate with sensitivity to HSP90 inhibition. (**a**) IC_50_ values for ganetespib in 16 cell lines (9 mesothelioma and 7 lung adenocarcinoma) have been correlated to the ability of the drug to downregulate MCL1, **P=0.0039. Number of cell lines downregulating MCL1 *n*=11, Number of cell lines not downregulating MCL1 *n*=5. (**b**) Explants derived form two mesothelioma patients were treated with ganetespib 2 μm for 24 h. Tissues have been stained with caspase 3 and MCL1. In patient #1, ganetespib induced apoptosis and downregulation of MCL1 (caspase 3 *P=0.0382; MCL1 **P=0.0069) and in patient #2, no significant apoptosis or MCL1 downregulation was observed. (**c**) MSTO-211H, H460 and H23 have been transfected with siMCL1 20 nm for 48 h. PARP cleavage and the expression of the pro-survival BCL-2 family members have been assessed by western blot. (**d**) MCL1 addiction has been tested in 16 cell lines and then correlated to the IC_50_ values for ganetespib; **P=0.0095. Number of cell lines addicted to MCL1 *n*=8 and Number of cell lines not addicted to MCL1 *n*=8.

HSP90 inhibitor-induced MCL1 downregulation correlated with sensitivity in a panel of nine mesothelioma and seven lung adenocarcinoma cell lines (Figure 3a, Supplementary Figure S4A). This association was also observed in live primary mesothelioma explants obtained at radical surgery (Figure 3b).

MCL1 silencing by RNA interference was sufficient to induce apoptosis (Figure 3c, Supplementary Figure S4B), implicating dependence to this pro-survival BCL-2 family protein. MCL1 silencing was conducted in thirteen additional cell lines (Supplementary Figure S4C) and two groups have been generated according to MCL1 addiction. MCL1-dependent cell lines were more sensitive to HSP90 inhibition (Figure 3d). To demonstrate that MCL1 downregulation following HSP90 inhibition correlates with MCL1 addiction, we used contingency analysis (*P*=0.0256) (Table 1). To establish whether MCL1 silencing induced cell death via the same repertoire of BH3-only proteins as HSP90 inhibition, we conducted a BH3-only protein focused RNAi screening and identified BIK and PUMA as essential. Apoptosis occurred in a caspase 8-independent manner (Supplementary Figure S5A–C).

### Ganetespib-resistant cells lose MCL1 dependence

**Figure 4:**
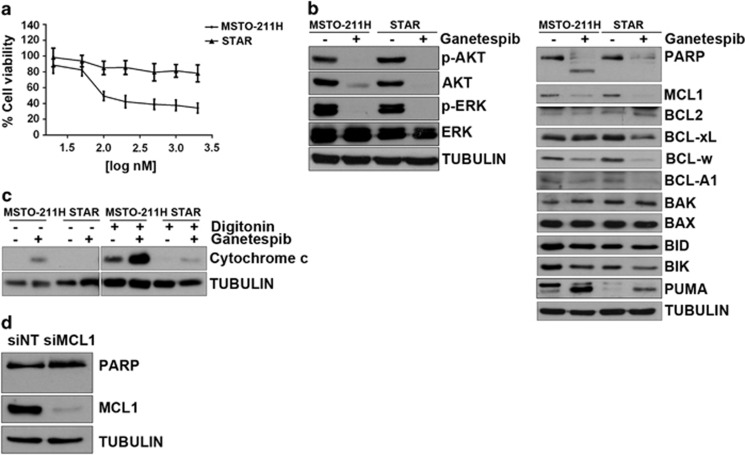
MCL1 dependence is lost in cells selected for resistance to ganetespib. (**a**) Cells selected for resistance (STAR) were tested for cell viability after 72 h treatment with ganetespib compared with parental MSTO-211H. (**b**) Parental and resistant cells were treated for 48 h with ganetespib 200 nm. On-target activity of ganetespib on Akt and ERK and the effect of HSP90 inhibition on pro- and anti-apoptotic proteins were analysed by western blot. (**c**) Cytochrome-c release was assessed after 48 h treatment with ganetespib 200 nm in the presence or absence of digitonin. Mitochondrial-free cytosolic fraction has been used for western blot analysis. (**d**) Resistant cells were transfected with siRNA targeting MCL1 for 48 h. Induction of apoptosis was measured by western blot with PARP antibody.

We then hypothesized that MCL1 suppression would itself be a selection pressure for acquired resistance leading to loss of either MCL1 downregulation or MCL1 dependence. We therefore generated MSTO-211H cells with greater than 1-logfold IC_50_ associated resistance to ganetespib (STAR) compared with isogenic parental cells, as evidenced by loss of viability and clonogenic assay (Figure 4a, Supplementary Figure S6A). Resistance was generalized to multiple HSP90 inhibitors, and these cells exhibited cross resistance to the endoplasmic reticulum stress inducer tunicamycin (Supplementary Figure S6B and C).

HSP90 inhibition still induced downregulation of MCL1 to the same extent in resistant compared with parental cells, as well as dephosphorylation of AKT and ERK (Figure 4b). Resistance was associated with a block in MOMP evidenced by a lack of cytochrome-c release (Figure 4c), and a lack of BAX/BAK-dependent caspase 8 activation (Supplementary Figure S6D). Although MCL1 downregulation occurred following HSP90 inhibition, the dependence of parental cells on this pro-survival protein changed such that MCL1 RNAi was no longer able to induce apoptosis alone (Figure 4d).

Genome-wide screen for copy number alterations revealed large structural variations throughout the genome; however, no specific selection for alterations in the loci encoding BCL-2 pro-survival genes was observed (Supplementary Figure S6E).

### Reversal of ganetespib resistance via concurrent pro-survival BCL-2 family inhibition

**Figure 5:**
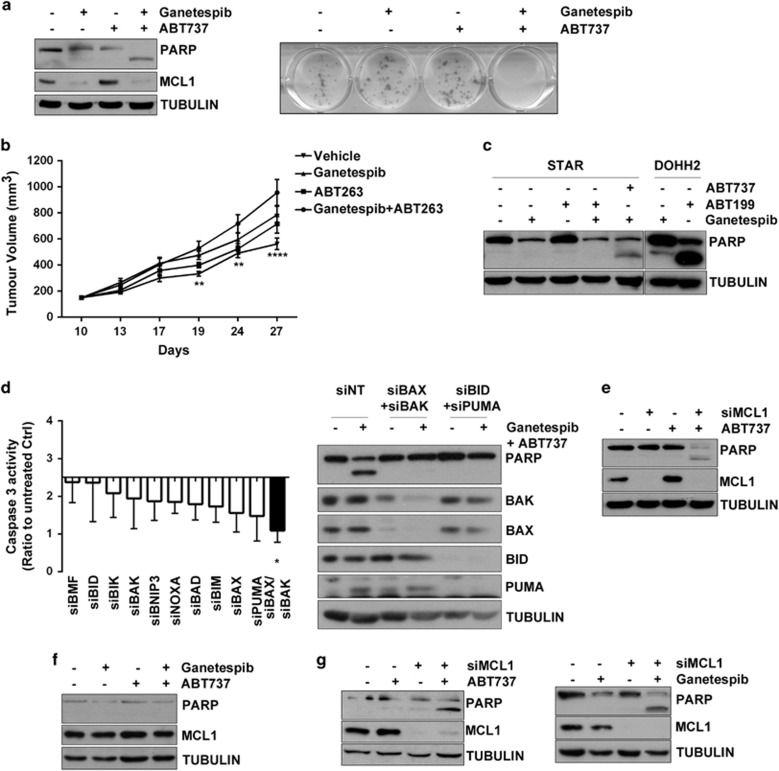
The combination of ganetespib and ABT737 overcomes acquired resistance through exploitation of MCL1 downregulation. (**a**) STAR cells were treated with ganetespib 200 nm, ABT737 200 nm or a combination of both for 48 h. PARP cleavage and MCL1 expression were measured by western blot. The effect on colony formation was measured by clonogenic assay. STAR cells were treated for 24 h with ganetespib 200 nm, ABT737 200 nm or a combination of both. After being washed, colonies were let to grow for 12 days, then fixed in methanol and stained with crystal violet. (**b**) Mice bearing established STAR tumours (*n*=7 per group) were dosed with vehicle, 100 mg/kg ABT263 (5 × /week), 100 mg/kg ganetespib (1 × /week), or the combination of ABT263 and ganetespib. Tumour volumes were assessed at end of the study. The combination of ABT263 with ganetespib resulted in a statistically significant decrease in tumour volume compared with vehicle control (error bars ±s.e.m.). (2-way Anova repeated measurements results: 19 days, ABT 263 + Ganetespib vs Vehicle, **P=0.0095; 24 days, ABT 263 + Ganetespib vs Vehicle, **P=0.0017; 27 days, ABT 263 + Ganetespib vs Vehicle, ****P<0.0001, ABT 263 + Ganetespib vs Ganetespib *P*=0.0132, ABT 263 + Ganetespib vs ABT 263 *P*=0.0004). The average body weight loss at end of the study was −7.1% with vehicle, −9.1% with ganetespib, −12.1% with ABT263 and −9.6% for the combination. (**c**) STAR cells were treated with ganetespib 200 nm, ABT199 200 nm, or a combination of both for 48 h. A combination of ganetespib and ABT737 in STAR and ABT199 in DOHH2 were used as a positive control. PARP cleavage and MCL-1 expression were measured by western blot. (**d**) STAR cells have been transfected with siRNAs targeting BH3-only protein. Twenty-four hours after transfection, cells have been treated with ganetespib 200 nm and ABT737 200 nm for further 48 h and caspase 3 activity measured. Data were normalized to siNT control (*P=0.0146). PARP cleavage, BAX, BAK, BID and PUMA expression was assessed by western blot. (**e**) STAR cells were transfected with siRNA targeting MCL1. Twenty-four hours after transfection, cells were left untreated or treated with ABT737 both for further 48 h. PARP cleavage and MCL1 expression were measured by western blot. (**f**) NCI-H28 cells were treated with ganetespib 200 nm, ABT737 200 nm or a combination of both for 48 h. PARP cleavage and MCL1 expression were measured by western blot. (**g**) NCI-H28 cells were transfected with siRNA targeting MCL1. Twenty-four hours after transfection, cells were left untreated or treated with either ABT737 or ganetespib for further 48 h. PARP cleavage and MCL1 expression were measured by western blot.

MCL1 has been reported to mediate resistance to the BCL-2, BCL-xL, BCL-w inhibitor, ABT737 and this resistance can be reversed by suppression of MCL1.^28^ We therefore hypothesized that in the acquired resistance setting, conserved transcriptional suppression of MCL1 might be exploited to sensitize to ABT737. Accordingly, a combination of ABT737 and ganetespib led to induction of cell death as shown by PARP cleavage, and significant reduction in colony formation (Figure 5a). This effect was also observed *in vivo* (Figure 5b). BCL-2 inhibition alone was insufficient to mediate this effect as evidenced by resistance to the combination of ganetespib with the BCL-2-specific inhibitor ABT199 (Figure 5c).

Using BH3-only protein focused RNAi, we observed that apoptosis induced by ABT737 and ganetespib in resistant cells required BAX/BAK and BID/PUMA (Figure 5d). MCL1 RNAi phenocopied ganetespib by inducing apoptosis when combined with ABT737 (Figure 5e). To corroborate these data, we performed a rescue experiment transfecting MCL1. The overexpression of MCL1 partially reduced the effect of the combination of ganetespib and ABT737 (Supplementary Figure S7A), with greater impact when RNAi targeting the 3' untranslated region of endogenous MCL1 was combined with ABT737 (Supplementary Figure S7B).

We then studied the effect of the combination treatment in the context of intrinsic resistance to examine whether ABT737 might also potentiate HSP90 inhibition induced apoptosis. MCL1 was not downregulated in NCI-H28 cells after treatment (Supplementary Figure S4A) and these cells were not addicted to MCL1 (Supplementary Figure S4B). Accordingly, combination with ABT737 did not reverse HSP90 inhibitor resistance (Figure 5f); however, treatment with ABT737 or ganetespib following MCL1 silencing did induce cell death (Figure 5g), confirming that MCL1 downregulation is necessary to activate apoptosis in this HSP90 inhibitor resistant setting.

## Discussion

**Figure 6:**
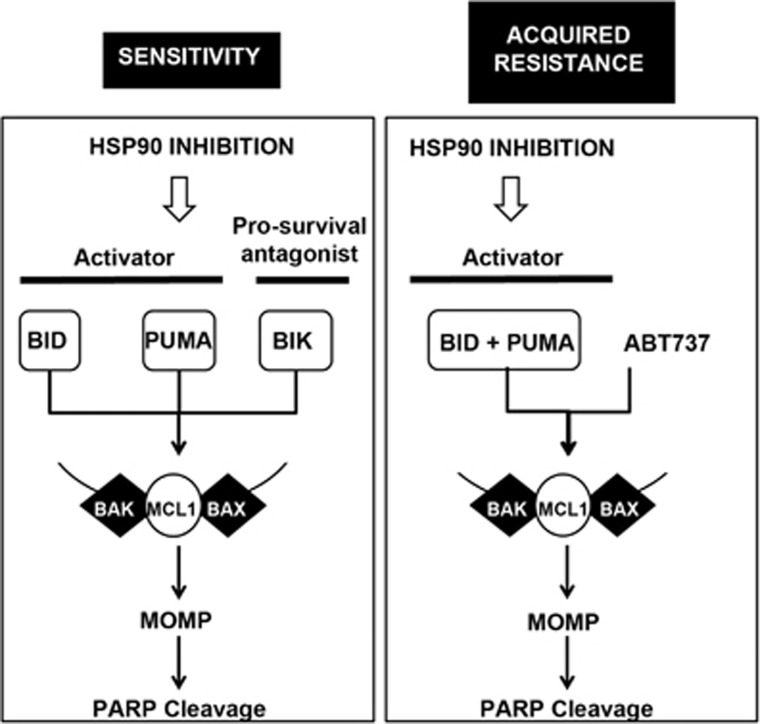
Schematic representation of the HSP90 inhibition-induced apoptosis in sensitive cells and in the context of acquired resistance. In sensitive cells, HSP90 inhibition targets the BH3-only proteins BID, BIK and PUMA and the pro-survival BCL-2 family protein MCL1 (white boxes). HSP90 inhibition requires complex interplay of these proteins to mediate BAX/BAK-dependent apoptosis. In resistant cells, MCL1 repression is conserved, however, BIK is not essential. To achieve apoptosis both the activation of BID and PUMA by HSP90 inhibition, and the targeting of the pro-survival proteins BCL-xL and BCL-w by ABT737, are needed.

Apoptosis block is a hallmark of cancer and may contribute to the onset of drug resistance.^29^ We have shown that apoptosis induced by inhibition of HSP90 involves the mitochondrial pathway and is activated by the reciprocal regulation of specific pro-apoptotic and anti-apoptotic BCL-2 family proteins. We have found that up to three BH3-only proteins (BID, BIK and PUMA) act in a coordinated manner, to trigger BAX/BAK-dependent cell death (Figure 6). This contrasts with single BH3-only protein dependence in the targeting of epidermal growth factor receptor, where BIM alone is required to induce cell death.^30, 31, 32^ This implies that the HSP90 inhibitor simultaneously damages multiple cell compartments leading to activation of p53-dependent PUMA transcription,^33^ BIK that signals to the endoplasmic reticulum^23^ and BID, which is activated by caspase 8 cleavage.^34^

We observed a requirement of BAX and BAK to mediate cell death in response to HSP90 inhibition, with a significantly reduced response in BAX- or BAK-negative models. This is in accordance with recent data showing that BAX is required for the induction of cell death after treatment with the HSP90 inhibitor 17-AAG.^24^ In the absence of BAX and BAK, caspase 8 cleavage was not observed in response to HSP90 inhibition, suggesting that cell death is mediated via the intrinsic pathway and caspase 8 is activated only as the result of a mitochondrial amplification loop.

A significant decrease in MCL1 expression was observed after treatment with HSP90 inhibitors *in vitro* and in explants from mesothelioma and this correlated with sensitivity to ganetespib. Focal amplification of MCL1 (1q21.2) has been reported as one of the most frequent copy number variation across human cancers and this correlates with addiction to MCL1 *in vitro*.^35^ We found that MCL1 dependence is associated with higher sensitivity to HSP90 inhibition, suggesting that a possible correlation with 1q21 amplification could be predictive for HSP90 inhibitors.

MCL1 has a short half-life of 30 min^36^ and can be rapidly regulated at transcriptional,^37^ post-transcriptional^38^ and post-translational levels.^39^ A conserved STAT5A binding site has been identified in both the promoter region and the 3' untranslated region of *Mcl1* in mice natural killer cells.^40^ We have shown for the first time that HSP90 inhibition reduces MCL1 luciferase reporter activity and mRNA expression, through interference with STAT5A-dependent activity. These findings are consistent with the previous reports in which inhibition of STAT3/5 can downregulate MCL1 and can induce apoptosis in response to tyrosine kinase inhibition.^41, 42^ We observed a failure to downregulate MCL1 in HSP90 inhibitor-resistant NCI-H28 cells and this was associated with failure to target STAT5 by an unknown mechanism.

In the acquired resistant context, MCL1 downregulation persisted alongside other markers of HSP90 inhibition, including inhibition of AKT and MAPK signaling. This suggested that selection did not involve loss of on-target activity, but rather, resistance occurred downstream of the HSP90-client interaction at the level of the cell death machinery. ABT-737 inhibits BCL-xL, BCL-w and BCL-2 and its apoptosis inducing efficacy is prevented by MCL1.^43^ As HSP90 inhibitor-resistant cells conserved MCL1 downregulation, we found that apoptosis could be re-activated by combining the HSP90 inhibitor with sub-lethal concentrations of ABT737. The apoptotic mechanism for this synergistic interaction utilized the same BH3-only proteins (BIK and PUMA) as for the HSP90 inhibitor alone in the parental cells; however in this context, the BH3-only protein BIK became redundant (Figure 6). Although a combination of MCL1 RNAi and ABT737 or ganetespib could induce apoptosis in intrinsically resistant NCI-H28 cells, this was not observed following HSP90 inhibitor plus ABT737, implying an MCL1-dependent mechanism. NCI-H28 cells had a block of MCL1 downregulation secondary to the lack of effect of HSP90 inhibition on STAT5A.

We have chosen to focus on thoracic cancer models as these are presently the focus of our ongoing trials. It is possible that the link shown between MCL1 and HSP90 inhibition can be more widely generalized, but this will require future studies to confirm. Results from the Phase IIB Galaxy1 trial in patients with metastatic lung adenocarcinoma, comparing the HSP90 inhibitor ganetespib and docetaxel with docetaxel alone, has shown that patients refractory to standard chemotherapy (<6 months) do not benefit from the combination treatment with ganetespib plus docetaxel (NCT01348126).^44^ Consistent with this, we observed that chemotherapy-resistant cell lines selected *in vitro* for cisplatin^45^ and vinorelbine^46^ exhibited cross resistance to HSP90 inhibition with loss of MCL1 dependence (Supplementary Figure S8).

In summary, even in the acquired resistance context, HSP90 inhibitors persist in downregulating MCL1 and can switch MCL1 dependence. This is exploitable via a synthetic lethal combination with ABT737 providing a rational strategy to bypass resistance.

## Materials and methods

### Reagents and antibodies

Ganetespib was obtained by Synta Pharmaceuticals (Lexington, MA, USA). ABT737 and ABT199 were kindly donated by Dr Vogler (University of Leicester, Leicester, UK). PU-H71 and Radicicol were purchased from Tocris Bioscience (Bristol, UK). 17-AAG was purchased from Sigma (St. Louis, MO, USA). The antibodies against PARP, BID, BIK, PUMA, CASPASE 8, BCL-2, BCL-Xl, BCL-w and GFP were obtained from Cell Signaling (Danvers, MA, USA), BAX, BAK and MCL1 antibodies were purchased from Santa Cruz Biotechnology (Dallas, TX, USA) and β-tubulin was obtained from Abcam (Cambridge, UK). Cytochrome-c antibody was purchased from BD PharMingen (Oxford, UK). Secondary antibodies were goat anti-rabbit HRP (DAKO, Glostrup, Denmark) and donkey anti-mouse HRP (GE Healthcare, Amersham, UK).

### Cell lines

Mesothelioma cell lines: ONE58, JU77, H2591 and H2461 were kindly provided by Dr PW Szlosarek, Institute of Cancer at Barts, London, UK); MM98 was kindly provided by Dr S Biffo, San Raffaele Scientific Institute, Milan, Italy). NCI-2452, NCI-2052, NCI-H28 and MSTO-211H were purchased from ATCC (Middlesex, UK). NSCLC cell lines: H460, H23, H1299, H1975 and NCI-H727 were purchased from ATCC; NCI-3122 was obtained from the NCI Tumour Repository. WT and BAX/BAK double knockout (DKO^BAX/BAK^) mouse embryonic fibroblasts were a kind gift from Dr Scott Oakes, University of California, San Francisco, USA). All cells lines were grown in RPMI Medium 1640, l-Glutamine and 10% FBS. Ganetespib-resistant MSTO-211H was generated by increasing exposure to ganetespib in a stepwise manner over duration of 8 months. Cells were incubated with 200 nm ganetespib for 72 h and then washed. Surviving cells were allowed to grow to confluence and then treated with increasing concentrations of ganetespib up to 2 μm.

### Measurement of cell viability and apoptosis

In all, 5000 cells per well were seeded in 96-well plate and incubated for 72 h in the presence or absence of HSP90i at concentrations ranging from 20 nm to 2 μm. Cell viability was assessed by MTT (Sigma) assay. For the Caspase 3 luminescence assay, cells were left untreated or incubated with 200 nm ganetespib. Forty-eight hours following treatment, cells were analysed using the Caspase-Glo 3/7 Assay protocol (Promega, Southampton, UK).

### Protein extraction and immunoblotting

Forty-eight hours after treatment cells were lysed in RIPA buffer containing protease inhibitors (Roche, Burgess Hill UK) and whole-cell lysates were clarified by centrifugation. In all, 40 μg of total cell lysates was loaded and separated on SDS-PAGE denaturing gels, transferred onto nitrocellulose membranes, and blocked in 5% milk-PBS-0.1% Tween. Membranes were probed with primary antibodies diluted in 5% milk-PBS-0.1% Tween-20 at 4 °C overnight. Signal detection was performed with ECL-plus chemiluminescent system (GE Healthcare).

### Flow cytometry

Samples were analysed on a BD FACS Calibur flow cytometer machine, using Cell Quest Pro software (Becton Dickinson, Oxford, UK). Cell death was determined after 48 h of treatment with ganetespib 200 nm by using propidium iodide (Sigma) staining to evaluate the percentage of cells with sub-diploidal DNA content.

### siRNA transfections

The non-silencing control NT, MCL1 sip53, siSTAT5A and BH3-protein targeting siRNAs were obtained from Qiagen (Valencia, CA, USA).

The sequences are MCL1 target sequence: CCCGCCGAATTCATTAATTTA, p53 target sequence: AAGGAAATTTGCGTGTGGAGT, STAT5A target sequence: AGGCACGTGGAGGAACTCTTA, BAD target sequence: ACGAGTTTGTGGACTCCTTTA, BID target sequence: CAGGGATGAGTGCATCACAAA, BIK target sequence: ACCACACTTAAGGAGAACATA, BIM target sequence: CGGAGACGAGTTTAACGCTTA, BMF target sequence: CTGGAGGATGATGTGTTCCAA, BNIP3 target sequence: AAGATACCAACAGGGCTTCTG and PUMA target sequence: CAGCCTGTAAGATACTGTATA.

Additional sequences for MCL1 (target sequence: CGAAGGAAGUAUCGAAUUU), BID (target sequence: GUAACUAACUGCAUACACU), BIK (target sequence: GGAGGGCAGUGACGCAUUG) and PUMA (target sequence: GUAGAUACCGGAAUGAAUU) were obtained from Dharmacon (Chicago, IL, USA).

siRNA transfections (20 nm) were performed using the RNAiMAX transfection reagent (Invitrogen, Carlsbad, CA, USA) according to the manufacturer's instructions.

### Overexpression of BAX

Mouse embryonic fibroblast DKO^BAX/BAK^ cells were transiently transfected with 1 μg of a GFP-tagged BAX construct or GFP empty vector, using Xtreme gene transfection reagent (Roche) according to the manufacturer's instructions.

### Real-time quantitative RT-PCR

Total RNA was extracted using Trizol (Invitrogen) according to the manufacturer's instructions. Reverse transcription was performed with High Capacity RNA-to-cDNA Kit (Applied Biosystem, Foster City, CA, USA). Real-time PCR was carried out using Power SYBR Green PCR Master Mix (Applied Biosystem) after 24 h of silencing or 48 h of treatment. QuantiTect primer assays (Qiagen) were used for MCL1 and Actin.

### MCL1 promoter sub-cloning

The full-length MCL1 promoter (352 bp)^47^ and the pGL2 basic empty vector were kindly donated by Prof. El-Tanani (Centre for Cancer Research and Cell Biology, Queens University Belfast, Belfast, UK). Three fragments of 277 bp, 193 bp and 115 bp have been generated by PCR and directional cloning with *Xho*I and *Hin*dIII (New England Biolabs, Ipswich, MA, USA) restriction sites. PROMO analysis^48^ was carried out on the three fragments to identify putative transcription factors binding sites.

### Reporter assay

Cells were transfected with pGL2 basic or pGL2-MCL1 and Renilla by using the Xtreme gene transfection reagent (Roche) according to the manufacturer's instructions. At time of transfections, cells had also been treated with HSP90i. Twenty-four hours after transfection cells were lysed and stored at −80 °C for at least 24 h. The luciferase activity was then measured by a Dual-Luciferase reporter assay system (Promega). Luciferase activity was normalized to Renilla activity.

### Explants

Primary pleural tissue was sectioned into small fragments (~64 mm^3^). Tissue explants were cultured in DMEM, 2% fetal calf serum, Penicillin Streptomycin, 2 mM Glutamax, allowed to recover overnight and then treated for 24 h with ganetespib 2 μm. In all, 5-μm sections were used for immunohistochemistry, as previously described.^49^ Primary antibodies were diluted in 1% goat serum/0.1% BSA/PBS (Cleaved Caspase 3, Cell Signaling, 1:200; MCL1, Santa Cruz Biotechnology, 1:150). The Rabbit-specific HRP/DAB (ABC) Detection IHC Kit (Abcam) has been used for the immunohistochemistry, according to the manufacturer's instructions. Sections were counterstained with haematoxylin and mounted using Vectamount permanent mounting media (Vector Labs, Peterborough, UK). Images were taken at × 40 magnification on a Hamamatsu Nanozoomer Digital slide scanner (Hamamatsu, Welwyn Garder City, UK). Appropriate ethical approval was obtained from the local research ethics committee to carry out this work.

### Scoring

For MCL1 as all cells stained evenly, only the intensity of the staining was measured on a scale 1–4. Cleaved Caspase 3 reflecting cell death events was scored as percentage of cells with cytoplasmic staining.

### Xenografts

Female CB.17 (SCID) mice (Charles River Laboratories, Wilmington, MA, USA) at 7–12 weeks of age were maintained in a pathogen-free environment and all *in vivo* procedures were performed in strict accordance with the NIH Guide for the Care and Use of Laboratory Animals and approved by the Synta Pharmaceuticals Corp. Institutional Animal Care and Use Committee. STAR cells were cultured as described and subcutaneously implanted into SCID mice at 5 × 10^6^ cells per animal following suspension in 50% matrigel (BD Biosciences, Oxford, UK). Mice bearing established tumours were allocated into treatment groups of seven exhibiting similar average tumour volumes (150 mm^3^) and dosed intravenously with vehicle (DRD; 10% dimethyl sulphoxide, 18% Cremophor RH 40, 3.6% dextrose) or ganetespib, or orally with ABT263, using the doses and schedules indicated. Ganetespib was formulated in DRD; ABT263 in PEG400/Ethanol/Phenthol as previously described.^50^ Tumour volumes (*V*) were calculated by caliper measurements of the width (*W*), length (*L*) and thickness (*T*) of each tumour using the formula: *V*=0.5236(*LWT*). Statistical analyses between the groups at end point were conducted using two-way ANOVA with repeated measurements (GraphPad Prism).

### Cytochrome-c release

Cells were treated with ganetespib 200 nm for 48 h and incubated in lysis buffer containing digitonin (Sigma) 0.0125% for 10 min, as previously described.^51^

### Oncoscan analysis

DNA was extracted with the QIAmp DNA Mini Kit (Qiagen). In all, 80 ng of gDNA was analysed using the OncoScan FFPE Assay Kit (Affymetrix, Wooburn Green High Wycombe, UK). Nexus Express Software for OncoScan 3.0.1 (Biodiscovery, Hawthorne, CA, UK) was used to carry out the analysis. Sample 1 was re-centred using the following region: chr1:64884261–113254220, chr6:99510013–147879973, chr7:65852764–159138663, chr12:75057068–123427028, chr14:37220996–85590956, chr17:662565–6708810 and chr19:51831082–57877327. Sample 2 was re-centred using the following regions: chr1:56680628–105050588, chr3:102941721–108987966, chr7:70656908–82749398, chr14:63600510–87785490, chr18:37427358–61612338

### Statistical analysis

Dose–response curves were fitted using non-linear regression (GraphPad Prism version 6.0, GraphPad Software, LaJolla, CA, USA). All data are representative of the median of three independent experiments. To determine correlation between sensitivity and MCL1 downregulation, MCL1 addiction we used the Mann–Wittney test. The Wilcoxon test was performed on all immunohistochemistry data. The Fisher's exact test has been used to analyse the contingency table data. The significance of the other data has been assessed with *t*-test or one-way ANOVA. All *P*-values less than 0.05 were considered as significant.

## Figures and Tables

**Table 1:** Contingency analysis showing correlation between MCL1 downregulation following HSP90 inhibition and MCL1 addiction

*P=0.0256*	*MCL1 downregulation*	*No MCL1 downregulation*	*Total*
MCL1 addiction	8	0	8
No MCL1 addiction	3	5	8
Total	11	5	16

The values are the number of cell lines that fall into each category.
