# Supplementary material for: Resistance to HSP90 inhibition involving loss of MCL1 addiction
Source: Oncogene. 2015 Jun 22;35(12):1483–92. doi: 10.1038/onc.2015.213 (PMC4819782; doi:10.1038/onc.2015.213)
Supplement: Supplementary Information [file onc2015213x1.pdf]

S1. BAX and BAK are required for Hsp90 inhibition-induced apoptosis. (A) WT and DKO $^{BAX/BAK}$ cells were treated with ganetespib 200 nM for 48 hours. SubG1 population was measured by FACS analysis (p=0.0047). (B) Cells were treated with ganetespib 200 nM, PU-H71 500 nM, 17AAG 1 µM and Radicicol 2 µM for 48 hours. PARP cleavage and MCL1 expression were analysed by western blot. (C) H460 stably expressing shRNA targeting BAX and BAX were treated with concentrations of ganetespib ranging from 20 nM and 2 µM. Viability was assessed MTT assay after 72 hours. Cells were treated with ganetespib 200 nM for 48 hours. SubG1 population was measured by FACS analysis (p<0.0001) (D) MSTO-211H cells were transfected with siNT, siBAX, siBAK and the combination of siBAX and siBAK. 24 hours following transfection, cells were treated with ganetespib for a further 48 hours and the subG1 population measured by FACS analysis (siBAX p=0.0012; siBAK p<0.0001; siBAX/BAK p<0.0001). (E) MSTO-211H transfected with siBAX/BAK and H460 shBAX/BAK were treated with ganetespib 200 nM for 48 hours. Caspase 8 cleavage was assessed by western blot.

S2. The BH3-only proteins BID, BIK and PUMA mediate ganetespib-induced apoptosis. (A) MSTO-211H cells were transfected with siRNA targeting BID, BIK or PUMA for 24 hours and treated with ganetespib 200 nM for further 48 hours. PARP cleavage and silencing efficiency were measured by western blot. SubG1 population was measured by FACS analysis (BID p=0.0127; BIK p=0.0057; PUMA p=0.0019). (B) H460 cells were transfected with siRNA targeting BID and BIK for 24 hours and treated with ganetespib 200 nM for further 48 hours. PARP cleavage and silencing efficiency were measured by western blot. SubG1 population was measured by FACS analysis (BID p=0.0004; BIK p=0.0184). (C) H23 cells were transfected with siRNA targeting BID and BIK for 24 hours and treated with ganetespib 200 nM for further 48 hours. PARP cleavage and silencing efficiency were measured by western blot. SubG1 population was measured by FACS analysis (BID p=0.0001; BIK p<0.0001). (D) MSTO-211H cells were transfected with siRNA targeting BIM for 24 hours and treated with ganetespib 200 nM for further 48 hours. PARP cleavage and silencing efficiency were measured by western blot. (E) MSTO-211H, H460 and H23 were transfected with Dharmacon siRNA targeting BID, BIK or PUMA for 24 hours and treated with ganetespib 200 nM for further 48 hours. PARP cleavage and silencing efficiency were measured by western blot.

S3. HSP90 inhibition induces STAT5A-dependent MCL1 suppression (A) MSTO-211H, H460 and H23 have been treated with ganetespib 200 nM, PU-H71 500 nM, 17AAG 1 µM and Radicicol 2 µM for 48 hours. PARP cleavage and MCL1 expression were analysed by western blot. (B) MSTO-211H, were treated with ganetespib 200 nM in presence or absence of ZVAD 50 µM for 48 hours. PARP cleavage and MCL1 expression were measured by western blot. (C) Three fragments of the MCL1 promoter were generated by PCR and subcloned with XhoI and HindIII restriction enzymes. Fragment A= 277bp, Fragment B= 193bp and Fragment C=115bp. (D) The effect of the siRNA targeting STAT5A was measured by qRT-PCR on RNA extracted from MSTO-211H and H28. Data were normalized to untreated control (MSTO-211H: p=0.0225; H28: p=0.0065) (E) The effect of the siRNA targeting p53 was measured by western blot.

S4. MCL1 addiction correlates with response to Hsp90 inhibition.
(A) A panel of 13 cell lines has been treated with ganetespib 200 nM for 48 hours. PARP cleavage and MCL1 expression were analysed by western blot.
(B) MSTO-211H, H460 and H23 were transfected with Dharmacon siRNA targeting MCL1 for 48 hours. PARP cleavage and silencing efficiency were measured by western blot.

(C) A panel of 13 cell lines has been transfected with siMCL1 20 nM for 48 hours. PARP cleavage and MCL1 expression were analysed by western blot.

S5. MCL1 silencing induces mitochondrial apoptosis. (A) MSTO-211H cells were transfected with siRNA targeting the eight BH3-only proteins in presence or absence of siMCL1. Apoptosis was measured by a Caspase3/7 assay (siBIK+siMCL1 p=0.0175; siPUMA+siMCL1 p<0.0001). (B) MSTO-211H cells were transfected with siRNA targeting siMCL1in presence or absence of sicaspase 8. Apoptosis was measured by a Caspase3/7 assay (siMCL1 p=0.0082; siC8+siMCL1 n.s.). (C) PARP cleavage and MCL1 and caspase 8 expression was measured by western blot.

S6. Cells selected for resistance to ganetespib acquire cross-resistance to other agents. (A) MSTO-211H and STAR were treated for 24 hours with ganetespib 100 nM, 200 nM and 400 nM. After being washed, colonies were let to grow for 12 days, then fixed in methanol and stained with crystal violet. (B) Parental and resistant cells were treated for 48 hours with ganetespib 200 nM, PU-H71 500 nM, 17AAG 1 µM and Radicicol 2 µM. Induction of apoptosis with PARP antibody and MCL1 expression were measured by western blot. (C) Cells were treated with ganetespib 200 nM and tunicamycin 5 µg/ml for 5 hours. ATF3 mRNA levels were measured by qRT-PCR (p=0.0411), splicing of XBP1 was measured with specific primer by PCR. (D) Cells were treated with ganetespib 200 nM and TRAIL 10 ng/ml for 48 hours. Induction of apoptosis with PARP antibody and Caspase 8 expression were measured by western blot. (E) Whole genome screenshot from Copy Number Variation analysis in MSTO-211H and STAR cells. Significantly altered regions are indicated by an asterisk.

S7. The combination of ganetespib and ABT7373 overcomes acquired resistance through exploitation of MCL-1 downregulation (A) STAR cells were transfected with HA-EV and HA-MCL1 in presence of the combination of ganetespib and ABT737. 48 hours after treatment PARP cleavage and MCL1 expression were assessed by western blot and Caspase 3 activity was measured (p=0.0188) (B) STAR cells were transfected with HA-EV and HA-MCL1 in presence of a siRNA targeting MCL-1. 24 hours after transfection cells were left untreated or treated with ABT737 both for further 48 hours. PARP cleavage and MCL1 expression were assessed by western blot and Caspase 3 activity was measured (p=0.0026).

S8. Cells selected for resistance to ganetespib acquire cross-resistance to chemotherapy and l oss of MCL1 addiction accounts for cross-resistance to ganetespib
(A) MSTO-211H and STAR cells have been treated with Cisplatin 10 µM, Vinorelbine 65 nM, Doxorubicin 500 nM, Docetaxel 10 nM and Bortezomib 50 nM for 48 hours. Apoptosis was measured by a Caspase3/7 assay (Cisplatin n.s., Vinorelbine p<0.0001, Doxorubicin p<0.0001, Docetaxel p=0.0007, Bortezomib p=0.0133). B) MSTO-211H and MSTOVINRES cells have been treated with Vinorelbine 65 nM and ganetespib 200 nM for 48 hours. Apoptosis was measured by a Caspase3/7 assay (Vinorelbine p<0.0001, Ganetespib p=0.0018). PARP cleavage and MCL1 expression were analysed by western blot. C) H460 and H460CISRES cells have been treated with Cisplatin 10 µM and ganetespib 200 nM for 48 hours. Apoptosis was measured by a Caspase3/7 assay (Cisplatin p=0.0011, Ganetespib p=0.0044). PARP cleavage and MCL1 expression were analysed by western blot. D) MSTOVINRES and H460CISRES were transfected with siRNA targeting siMCL1. PARP cleavage and MCL1 expression were measured by western blot.
