# Supplementary figures and images for: Resistance to HSP90 inhibition involving loss of MCL1 addiction
Source: Oncogene. 2015 Jun 22;35(12):1483–92. doi: 10.1038/onc.2015.213 (PMC4819782; doi:10.1038/onc.2015.213)

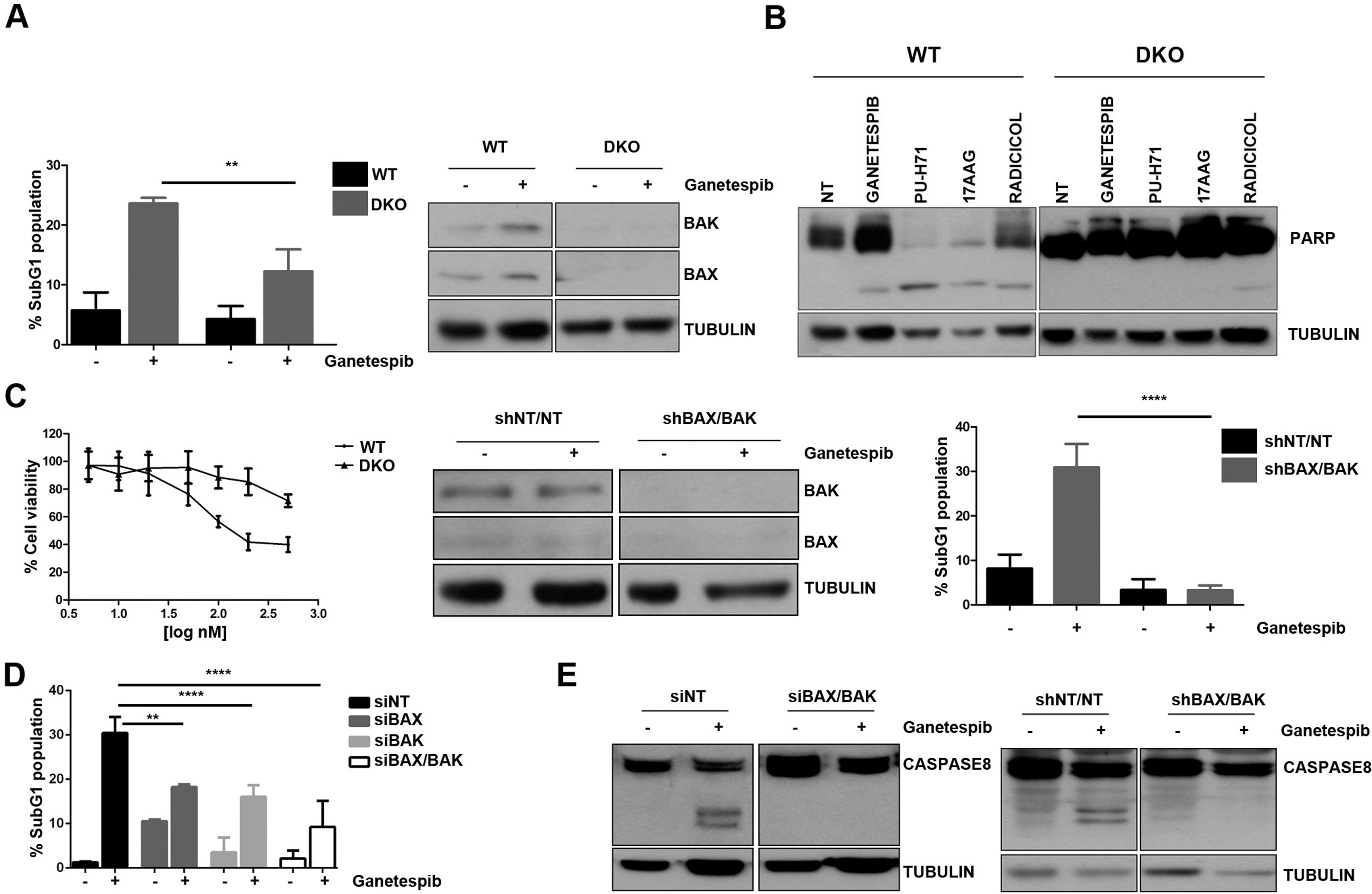

Supplement: Supplementary Figure 1 [file onc2015213x2.tif]

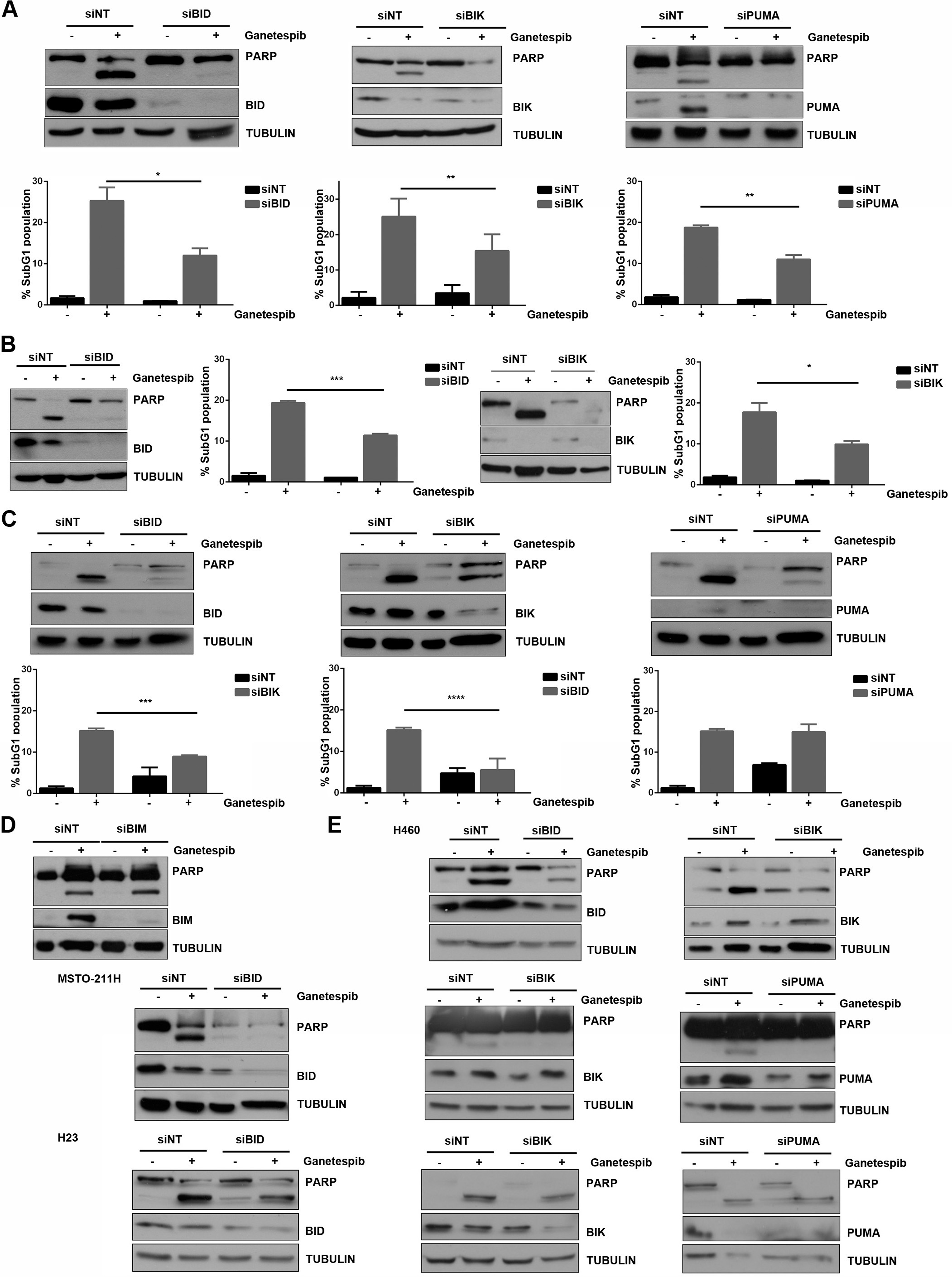

Supplement: Supplementary Figure 2 [file onc2015213x3.tif]

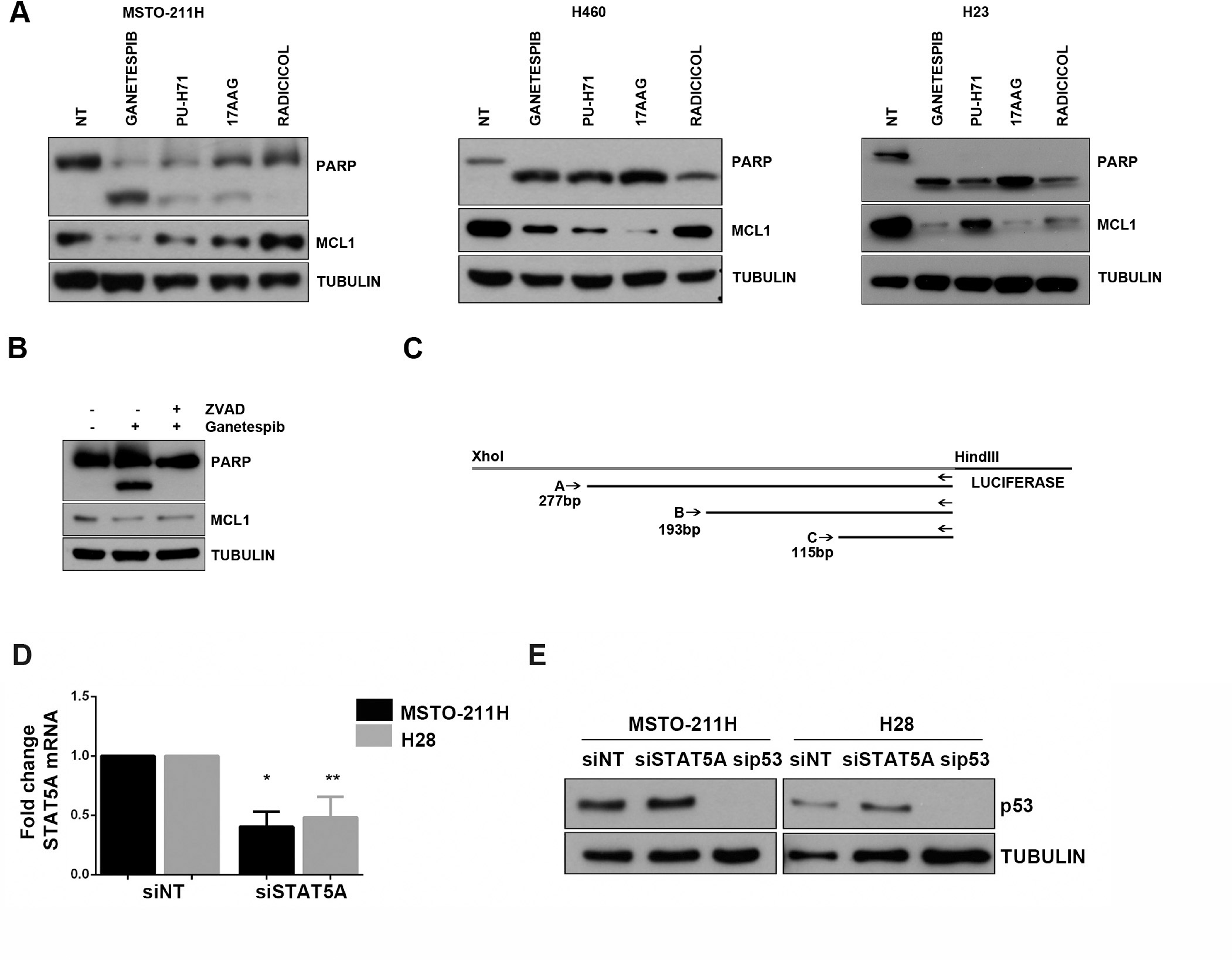

Supplement: Supplementary Figure 3 [file onc2015213x4.tif]

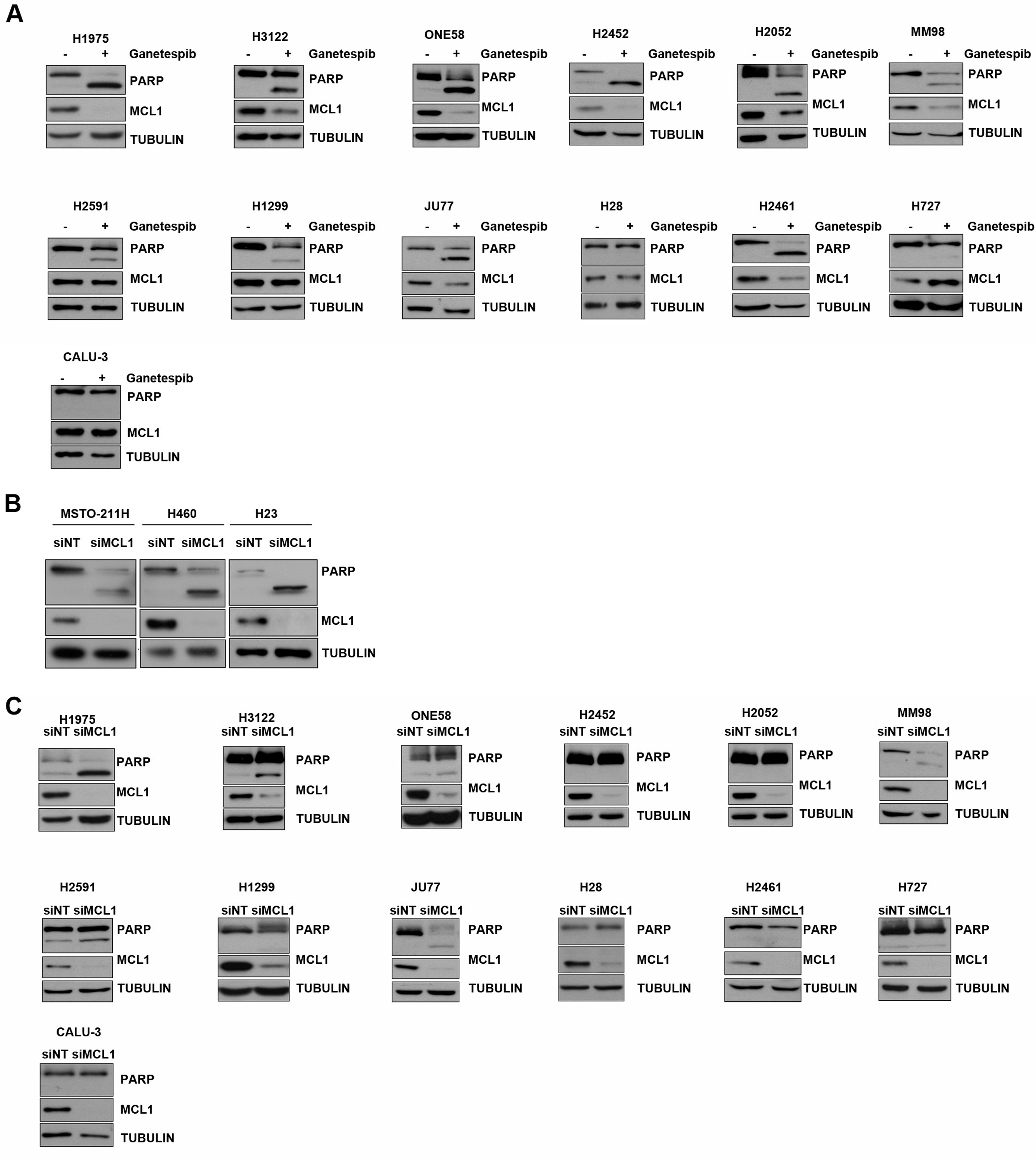

Supplement: Supplementary Figure 4 [file onc2015213x5.tif]

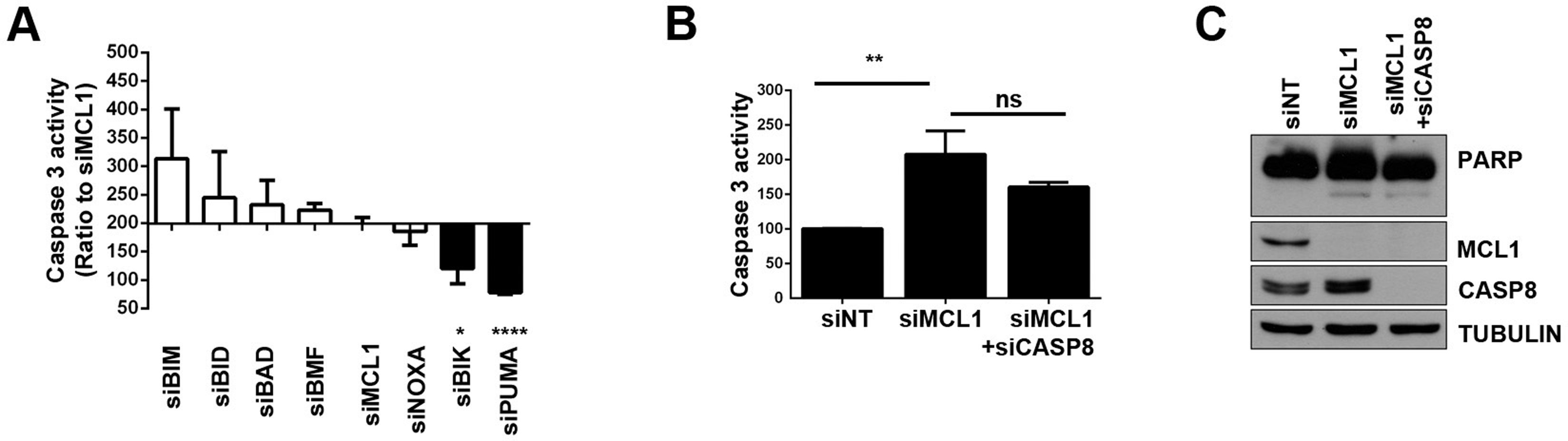

Supplement: Supplementary Figure 5 [file onc2015213x6.tif]

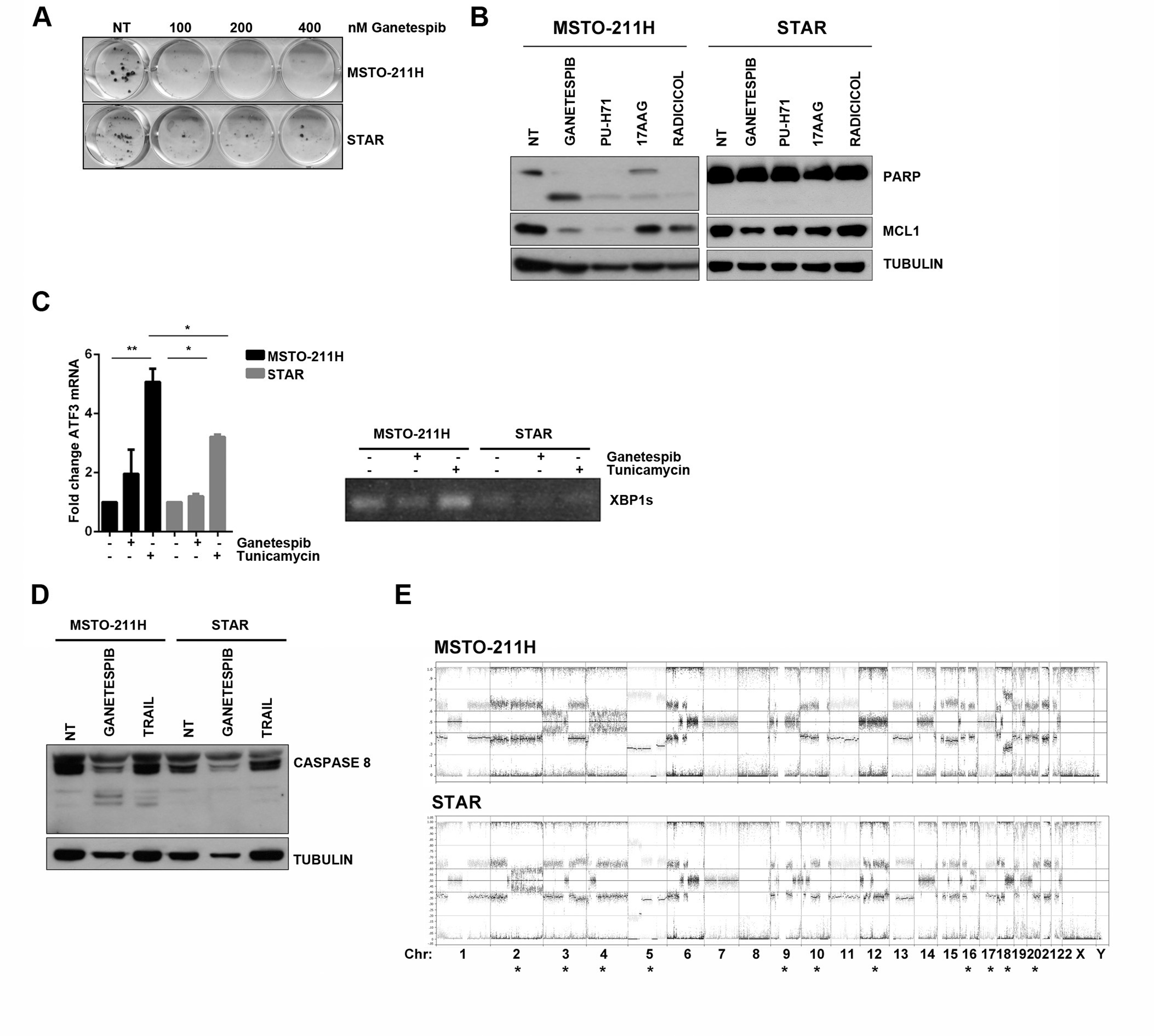

Supplement: Supplementary Figure 6 [file onc2015213x7.tif]

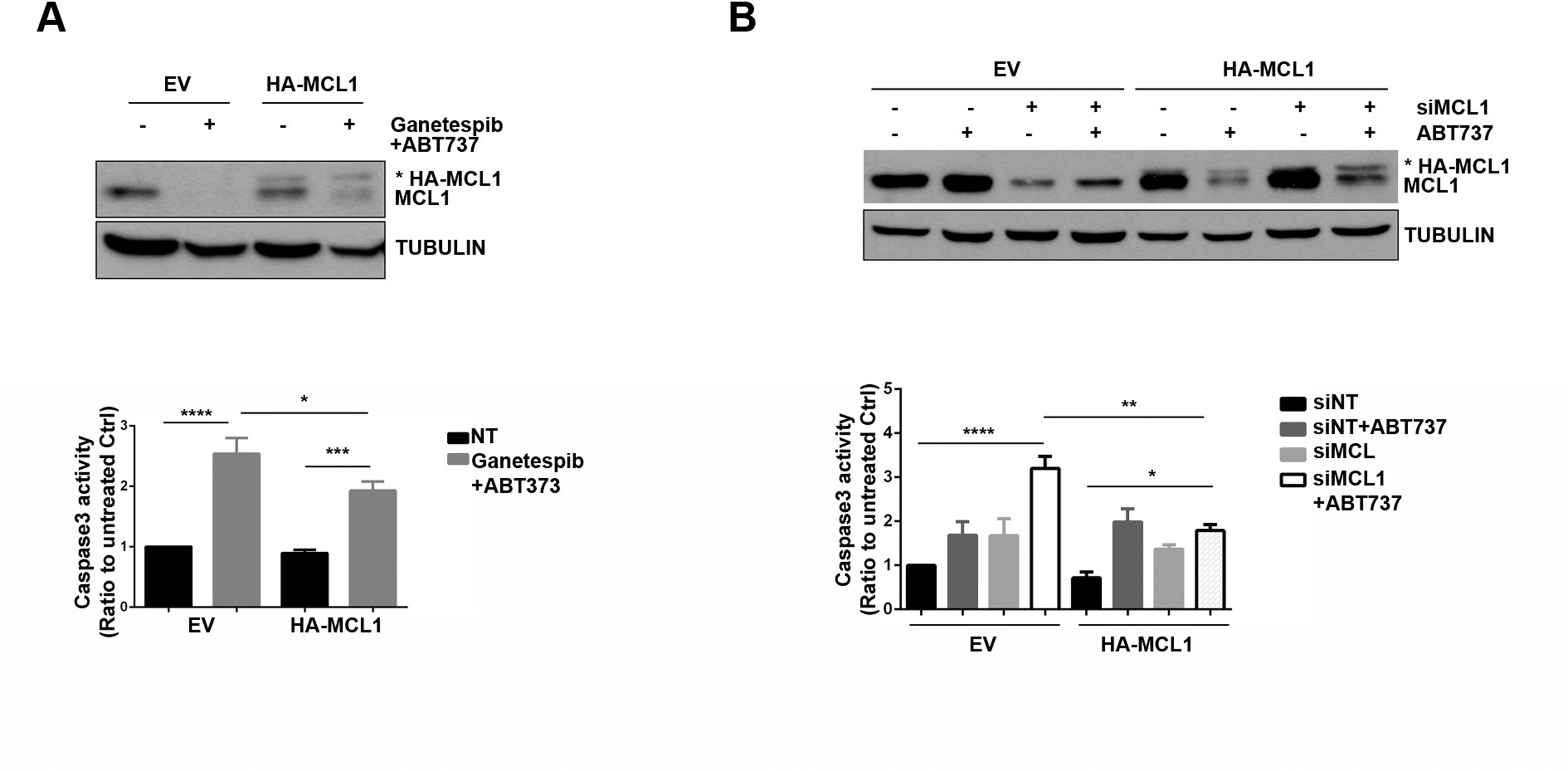

Supplement: Supplementary Figure 7 [file onc2015213x8.tif]

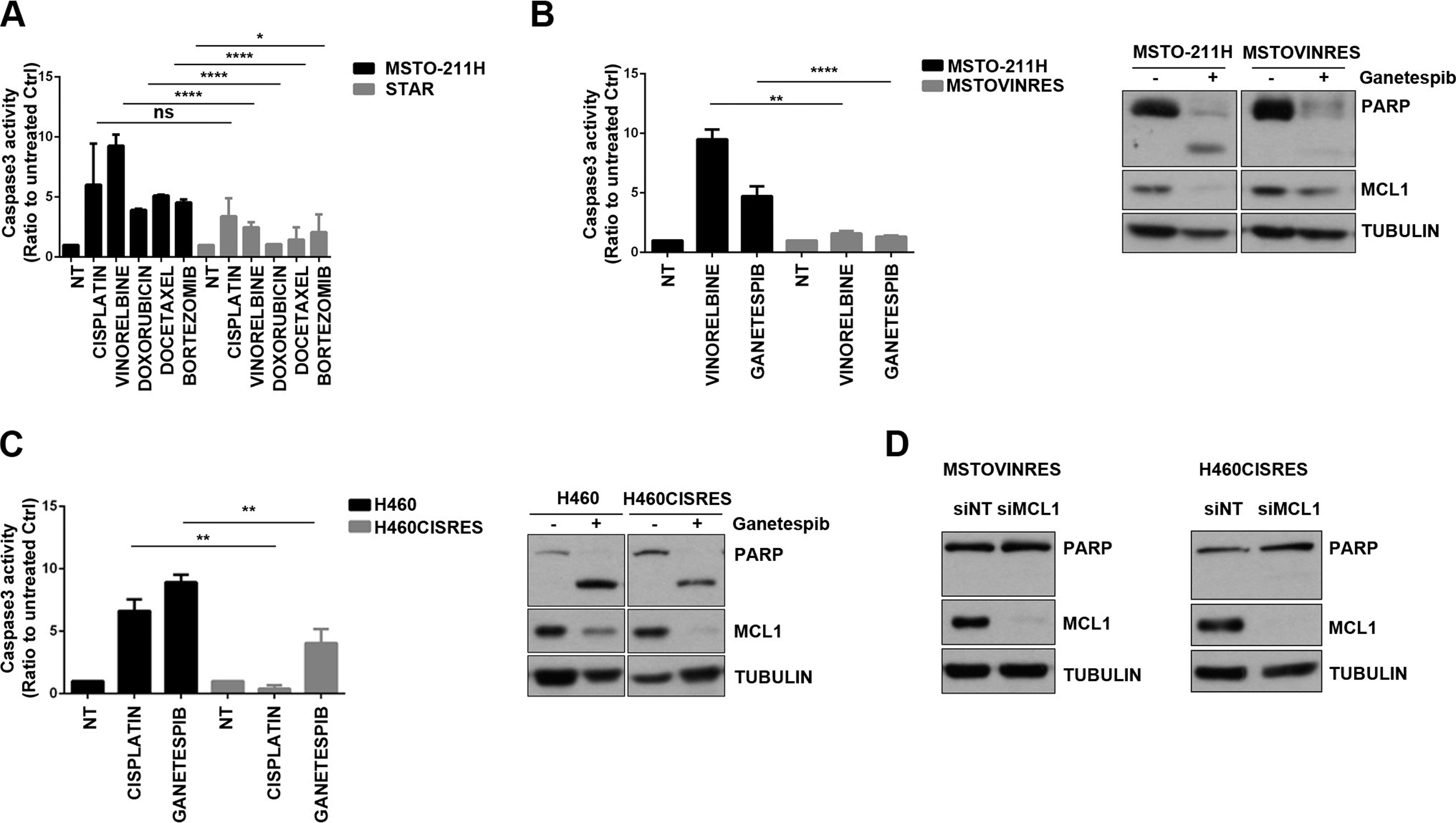

Supplement: Supplementary Figure 8 [file onc2015213x9.tif]
